# A Pooling Genome-Wide Association Study Identifies Susceptibility Loci and Signaling Pathways of Immune Thrombocytopenia in Chinese Han Population

**DOI:** 10.1155/2020/7531876

**Published:** 2020-03-11

**Authors:** Yanmei Xu, Jing Li, Wentao Yang, Xiaoli Tang, Bo Huang, Jing Liu, Jin Lin, Jing Zhang, Weiming Yang, Shuqi Li, Fan Sun, Libin Deng, Xiaozhong Wang

**Affiliations:** ^1^Department of Clinical Laboratory, Jiangxi Province Key Laboratory of Laboratory Medicine, The Second Affiliated Hospital of Nanchang University, Nanchang 330006, China; ^2^Department of Clinical Laboratory, The First Affiliated Hospital of Nanchang University, Nanchang 330006, China; ^3^Department of Organ Transplantation, The Second Affiliated Hospital of Nanchang University, Nanchang 330006, China; ^4^College of Basic Medical Science, Nanchang University, Nanchang 330031, China; ^5^Institute of Translational Medicine, Nanchang University, Nanchang 330031, China

## Abstract

Immune thrombocytopenia (ITP) is an acquired bleeding disease due to immune-mediated destruction of antilogous platelets and ineffective thrombopoiesis. Although the etiology of ITP remains unknown, genetic variants are thought to predispose individuals to the disease. Several candidate gene analyses have identified several loci that increased ITP susceptibility, but no systematic genetic analysis on a genome-wide scope. To extend the genetic evidence and to identify novel candidates of ITP, we performed a pooling genome-wide association study (GWAS) by IlluminaHumanOmniZhongHua-8 combining pathway analysis in 200 ITP cases and 200 controls from Chinese Han population (CHP). The results revealed that 4 novel loci (rs117503120, rs5998634, rs4483616, and rs16866133) were strongly associated with ITP (*P* < 1.0 × 10^−7^). Expect for rs4483616, other three loci were validated by the TaqMan probe genotyping assay (*P* < 0.05) in another cohort including 250 ITP cases and 250 controls. And rs5998634 T allele was more sensitive to glucocorticoids for ITP patients (*χ*^2^ = 7.30, *P* < 0.05). Moreover, we identified three overrepresented signaling pathways including the neuroactive ligand-receptor interaction, pathways in cancer, and the JAK-STAT pathway, which involved in the etiology of ITP. In conclusion, our results revealed four novel loci and three pathways related to ITP and provided new clues to explore the pathogenesis of ITP.

## 1. Introduction

Immune thrombocytopenia (ITP) is an autoimmune disorder characterized by low platelet count [1]. The incidence of adult ITP is about 5–10 cases/100,000 population annually in China [2]. However, the etiology of ITP is unclear and is considered multifactorial and polygenic in most cases. The research suggested genetic factor plays an important role in the pathogenesis of ITP [3]. Several susceptible genes of ITP have been identified by traditional candidate gene approaches including direct sequencing and polymerase chain reaction-restriction fragment length polymorphism (PCR-RFLP), but these mutations only explain a small fraction of ITP risk. The majority of heritability for ITP remains to be further elucidated.

Genome-wide association studies (GWAS) are a powerful tool in searching for gene variants of complex diseases by comparing single-nucleotide polymorphisms (SNPs) [4]. A number of repeatable susceptibility loci have been gradually translated into clinical treatment, prognosis, and pharmacological guidelines [5–8]. GWAS with pooled DNA has been widely used due to its rapid, efficient, and cost-effective performance [9].

To extend the present genetic data and to identify the novel genetic and biological functional evidence of ITP, we firstly performed a pooling GWAS in 200 ITP patients and 200 control subjects from CHP using an IlluminaHumanOmniZhongHua-8 array scanning 862,620 SNPs across the autosomal region. By SNP-Map (single-nucleotide polymorphism microarrays and pools) analysis, our scanning revealed 4 novel loci (rs117503120, rs5998634, rs4483616, and rs16866133) were strongly associated with ITP from CHP. Furthermore, we validated the relationship between rs117503120, rs5998634, and rs16866133 and ITP by the TaqMan probe genotyping assay (*P* = 0.0019). Moreover, we analyzed the relationship of loci and clinical therapy and found rs5998634 had a positive association with response to glucocorticoids (*χ*^2^ = 7.30, *P* = 0.03), suggesting that this SNP may have predictive value for the response to steroid treatment.

To provide further insight into the molecular function of these associated variants, we performed KEGG (Kyoto Encyclopedia of Genes and Genomes) pathway analysis based on the GWAS data. The most potential candidate pathways associated with ITP were the neuroactive ligand-receptor interaction, the pathways in cancer, and the JAK-STAT pathway.

In conclusion, our results suggest that these significantly associated loci, genes, and pathways may provide novel insights into the genetic etiology of ITP and novel clues for investigating the pathogenesis of ITP.

## 2. Materials and Methods

### 2.1. Patients and Controls

This study was carried out in accordance with the principles of the 1964 Declaration of Helsinki and its later amendments or comparable ethical standards and was approved by the Ethics Committee of the Second Affiliated Hospital of Nanchang University (No. Review [2010] No. (002)). All participants signed a written informed consent. A total of 450 adult ITP patients, who met the diagnostic criteria of consensus of Chinese experts on diagnosis and treatment of adult primary immune thrombocytopenia (version 2009, 10], from a Chinese Han population were enrolled during May 2010 to Feb 2017. None of the recruited ITP patients had hepatosplenomegaly or lymphadenopathy. In addition, the patients had normal or increased bone marrow megakaryocytes and significantly decreased peripheral blood platelet count. Familial ITP cases were not recruited in this study. In addition, patients with other types of thrombocytopenia such as heparin-induced thrombocytopenia or drug-induced thrombocytopenia were excluded. The 400 healthy unrelated control subjects were age- and sex-matched Chinese Han. Peripheral blood was collected from all participants including early-onset ITP cases and healthy controls. Clinical data from the two groups including platelet count (PLT), white blood cell (WBC), red blood cell (RBC), and hemoglobin (HB) were collected. The flowchart of two-stage sample collection was shown in Supplementary [Supplementary-material supplementary-material-1].

### 2.2. Response to Glucocorticoid Treatment

A total of 183 inpatients with ITP in the second stage were treated with glucocorticoids, including high-dose dexamethasone (HD-DEX) 40 mg daily for 4 days every 4 weeks and prednisone 1.0 mg/kg daily, which was then tapered. The enrolled patients were classified into two groups according to their response to glucocorticoid treatment: glucocorticoid response group (120 cases) and nonresponse group (63 cases). The response group included patients with complete response, which was defined as PLT higher than 100 × 10^9^/L, or with a partial response, which was defined as PLT ranging from 30–100 × 10^9^/L or at least doubling of the baseline count. The nonresponse group only contained patients without a response, which was defined as PLT lower than 30 × 10^9^/L or less than doubling of the baseline count. The criteria for complete response, response, and nonresponse were judged according to previous criteria [10].

### 2.3. Pooling GWAS

Genomic DNA was extracted from peripheral blood leukocytes from 200 cases and 200 controls using the Qiagen DNA Isolation kit (Qiagen DNA GmbH, Hilden, Germany) according to the manufacturer's protocol. Samples of intact genomic DNA showing no evidence of contamination by RNA and DNA degradation were selected for further analysis. DNA concentration and purity were calculated with a Nano Drop ND-2000 spectrophotometer (Nano Drop Technologies, DE, USA). For SNP-MaP scanning, the DNA concentration of each sample was quantified and adjusted to 5 ± 0.5 ng/*μ*L using DNase-free water. The compared pools consisted of 200 cases and 200 control subjects, respectively. DNA (5 *μ*L) of the case group was added to the case pool in equivalent molar amounts, and the same operation was applied for the control group. The concentration and purity of each pool were measured again. At last, eligible pools were processed for labeling and hybridization on IlluminaHumanOmniZhongHua-8 arrays according to the manufacturer's instructions (Illumina, Santa Clara CA, USA).

### 2.4. Analysis of SNPs

The hybridization intensities of two probes for each SNP allele were derived from the raw scanning files. The frequencies of autosomal SNPs were averaged over three replicate case and control arrays. Before analyzing the chip data, the quality control should be conducted, as mentioned in the following points: (1) due to the phenomenon of hybridization failure of a small number of sites will occur in the process of gene chip hybridization, the failed sites should be deleted; (2) considering that too few SNP detection experiments will lead to unreliable experimental results, it is necessary to remove the sites with less than 3 repetitions; and (3) the DNA pooling method mixes the whole genomic DNA of male and female patients, resulting in uneven loci on the extraordinary chromosomes (*X*, *Y*, and mitochondria), so the loci on the extraordinary chromosomes are not considered. Differences in allelic frequency between the sample pools were evaluated by combined *Z*-test, as follows:

(1) $$\begin{array}{c} T_{comb}=\frac{{\left({\bar{f}}^{\left(1\right)}-{\bar{f}}^{\left(2\right)}\right)}^{2}}{v_{1}+v_{2}+\varepsilon_{1}^{2}+\varepsilon_{2}^{2}}. \end{array}$$

The statistic combines

- Chi-square statistic, *T*_1_, for testing differences between two proportions (allele frequencies) in cases and controls accounting for sampling variance:

(2) $$\begin{array}{c} T_{1}=\frac{{\left({\bar{f}}^{\left(1\right)}-{\bar{f}}^{\left(2\right)}\right)}^{2}}{v_{1}+v_{2}}, \end{array}$$

where *f* = *Graw*/(*Graw* + *Rraw*) and represents the approximation of allele A frequencies for each replicate, averaged over the number of replicates in each pool (Graw and Rraw are the intensities of the green and red fluorescence value). ${\bar{f}}_{k}=1/n_{k}\sum_{i=1}^{n_{k}}f_{i}^{\left(k\right)}$ represents the allele frequencies over *n*_*k*_ pool replicates, $v_{k}={\bar{f}}_{k}\left(1-{\bar{f}}_{k}\right)/2N_{k}$ represents the binomial sampling variance, and *N*_*k*_ represents the number of controls and cases (*k* = 1, 2).

(b) *Z*-statistics for testing the differences in mean allele frequencies between cases and controls:

(3) $$\begin{array}{c} Z=\frac{\left({\bar{f}}_{1}-{\bar{f}}_{2}\right)}{\sqrt{\varepsilon_{1}^{2}+\varepsilon_{2}^{2}}}, \end{array}$$

where $\varepsilon_{k}^{2}=1/n_{k}\left(n_{k}-1\right)\sum_{i=1}^{n_{k}}{\left(f_{i}^{\left(k\right)}-{\bar{f}}_{k}\right)}^{2}$ is the square of the standard error.

Then, the *relative allele frequency (RAF)* of these two DNA pools was calculated using a method described previously [11]. For loci located nearby candidate genes, the false-positive report probabilities (FPRP) were calculated with the RAF to estimate the confidence intervals and the *P* value corresponding to the odds ratio (OR) scores [12].

The other SNP in linkage disequilibrium (LD) to the leading SNPs was analyzed using the functional mapping and annotation of genome-wide association studies (FUMA GWAS) tool (https://fuma. ctglab.nl/) [13].

### 2.5. TaqMan Probe Genotyping Analysis

The concentration of DNA samples from 250 cases and 250 controls was adjusted to 20 ng/*μ*L using DNase-free water. Genotyping was performed using TaqMan SNP Genotyping Assays (Life Technologies, USA), TaqMan Genotyping Master Mix (Life Technologies, USA), and an Applied Biosystems ViiA™ 7 Real-Time PCR System (Life Technologies, USA) in a 96-well format. The selected SNPs were genotyped with TaqMan® SNP Genotyping Assays: AHCTBKM for rs5998634, AHQJQ0L for rs17503120, and C_32336830_10 for rs16866133, except for rs4483616 due to the failure probe synthesis. Each reaction (10 *μ*L) contained 5.0 *μ*L TaqMan Genotyping master mix, 0.25 *μ*L primers and probes, 3.75 *μ*L DNase-free water, and 1.0 *μ*L DNA (20 ng/*μ*L). Thermal cycling conditions were 95°C for 10 min, followed by 40 cycles of 95°C for 15 seconds and 60°C for 1 min.

### 2.6. SNPs Mapping to Genes

To scan for the genetic factors related to ITP, all identified SNPs were mapped to genes utilizing the EntrezGene database (http://www.ncbi.nim.nih.gov/entrez/). Using the information of chromosome and position, we located SNPs to the genes within a window 20 Kbp upstream and downstream. The annotations for human genome assembly version 37 (Feb. 2009, hg19, GRCh37), which was downloaded from the UCSC genome annotation databases (http://hgdownload.cse.ucsc.edu/), was used to map SNPs to genes. In our study, if multiple SNPs were mapped to the same gene, only the gene with the lowest *P* value was selected for further analysis. If no gene was found in a +/-20Kbp window of the SNP, the nearest gene on each side of the SNP was included.

### 2.7. Pathway Analysis of GWAS Data

To provide further insight into the molecular function of identified associated variants, we utilized the WebGestalt (WEB-based Gene Set Analysis Toolkit, http://www.webgestalt.org/) to conduct functional enrichment analysis for genes in our study. All identified overrepresented pathways in our study derived from the KEGG pathway database (http://www.genome.ad.jp/kegg), which integrates genomic, chemical, and systemic functional information [14]. Although 7 genes reached the most significant criteria that *P* < 1.0 × 10^−7^, it was difficult to conduct a pathway analysis with such a few numbers of genes. Thus, we selected 287 genes by selecting *P* value less than 10^−5^ as a gene set to analyze. The term “hsapiens” was selected as the organism and “hsapiens_gene_symbol” as the gene ID type when uploading both the interesting gene list and the reference gene set. Then, “hsapiens_genome” was selected as the reference set, and *P* < 0.05 was considered as significant when the hypergeometric method was used for statistical analysis.

### 2.8. Statistical Analysis

In the clinical characteristics analysis, the continuous data were presented as mean ± standard deviation (SD), and the differences between cases and controls were evaluated using Student *t* tests or Chi-square tests where appropriate. *P* < 0.05 was considered statistically significant. In the discovery stage, the differences in allelic frequency between the sample pools were evaluated by combined *Z*-test. Statistical analysis was performed using SAS version 9.1.3 (SAS Institute Inc, Cary, NC, USA). Chi-square tests were used to detect whether the genotype distributions for the studied SNPs fit Hardy-Weinberg equilibrium (HWE), and *P* > 0.05 was considered to be consistent with the HWE equilibrium. Finally, 2 × 3 contingency tables were applied to compare genotype frequencies between cases and controls, and *P* < 0.05 was considered statistically significant.

## 3. Results

### 3.1. Clinical Characteristics

All of the recruited ITP patients demonstrated similar typical symptoms and signs. Two-hundred cases (65 males and 135 females) and 200 controls were recruited in the first stage. The mean age at the time of ITP onset was 40.36 ± 16.39 years, and the ratio of males and females was 1 : 2.1. A replicate cohort containing 250 cases (82 males and 168 females) and 250 unrelated controls was collected for the TaqMan probe genotyping assay. The mean age at the time of ITP onset was 40.85 ± 12.21 years, and the ratio of male to female was 1 : 2.04. Our data displayed a significant gender disparity. In both cohorts, PLT, RBC, and HB were significantly lower in the ITP group than in the control group (*P* < 0.001); however, WBC in ITP patients was significantly higher than in controls (*P* < 0.001). The detailed clinical information of all the subjects is shown in Table 1.

### 3.2. Loci Associated with ITP

**Figure 1:**
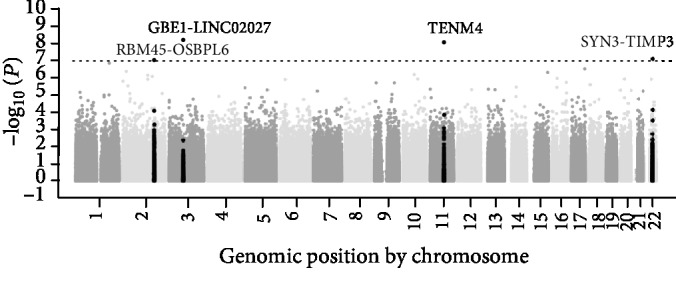
Pooled DNA-based GWAS results for ITP. The Manhattan plot of the GWAS shows the *P* value for the 862,620 autosomal SNPs. SNPs are plotted on the *x*-axis according to their position on each chromosome, and the negative log-10 of the *P* value was plotted on the *y*-axis. The horizontal dotted line indicates a *P* = 1.0 × 10^−7^, which is the criteria of selecting SNPs for GWAS.

**Figure 2:**
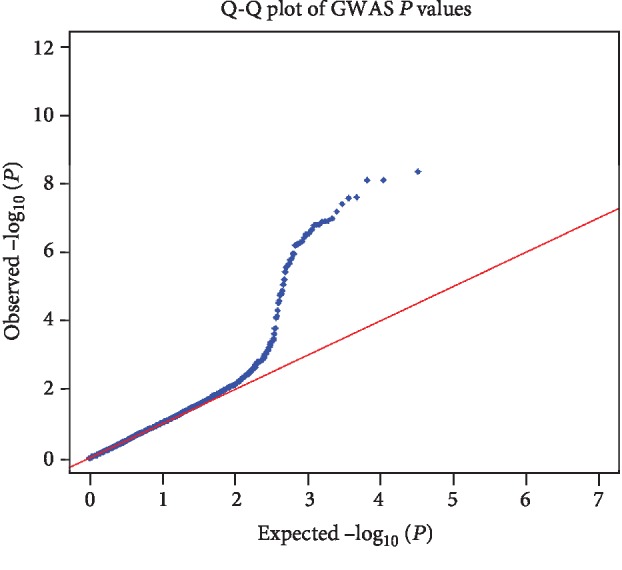
Quantile-quantile (Q-Q) plot of SNPs from ITP GWAS in Chinese Han population. *X*-axis is the expected value (-log_10_ (*P*)), and *Y*-axis is the actual observed value (-log_10_ (*P*)).

**Figure 3:**
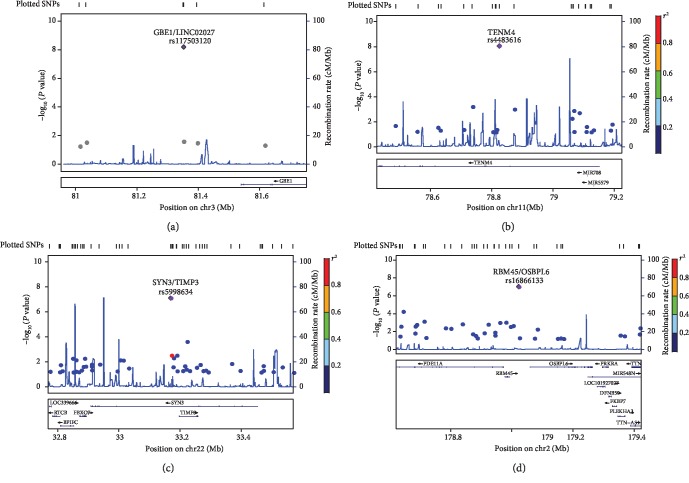
Regional plots of four significant loci. SNPs are plotted by position on chromosome against association with ITP (-log_10_ (*P* value)). The SNP name shown on the plot was the most significant SNP. The association between *GBE1/LINC02027*, *TENM4*, *SYN3/TIMP3*, *RBM45/OSBPL6*, and ITP in Chinese Han Population showed that the most significant site was rs117503120, rs4483616, rs5998634, and rs16866133, respectively, which were supported by the LD site (a–d). Plots were generated using LocusZooM (http://csg.sph.umich.edu/locuszoom).

For SNP-Map scanning, 862,620 autosomal SNPs were screened by the IlluminaHumanOmniZhongHua-8 arrays. The raw data were deposited in the Gene Expression Omnibus as data set GSE76744 (https://www.ncbi.nlm.nih.gov/geo/query/acc.cgi?acc=GSE76744). The averaged relative allele frequencies (RAF) of cases and controls presented a very high Pearson correlation (*r*^2^ = 0.9947, Supplementary [Supplementary-material supplementary-material-1]), indicating that the GWAS data was reliable for follow-up analysis. The difference in allelic frequency was assessed using the *Z* combination test, and the *P* value distribution is shown in Figure 1. The quantile-quantile (Q-Q) plots (Figure 2) presented that an excess of small *P* values compared to the distribution expected under the null hypothesis. According to the Bonferroni correction for multiple tests, the top four loci (rs117503120, rs4483616, rs5998634, and rs16866133) located within or near *GBE1*/*LINC02027*, *TENM4*, *SYN3*/*TIMP3*, and *RBM45*/*OSBPL6* genes reached a statistical significance in association with ITP in the genome-wide scale (*P* < 1.0 × 10^−7^, Figure 1, Table 2). And the regional plots of four significant loci in the corresponding genes were shown in Figure 3. To further measure the strength of SNPs associated with ITP, we calculated the odds ratio scores and FPRP for each high-ranking SNP. As shown in Supplementary [Supplementary-material supplementary-material-1], among the top 20 SNPs, only rs5998634 located within or near the genes *SYN3/TIMP3* showed an FPRP value of less than 1.0 × 10^−5^. The other SNPs in linkage disequilibrium (LD) to the top four loci were shown in Supplementary [Supplementary-material supplementary-material-1].

### 3.3. Validation of SNPs Associated with ITP

To confirm the association between significant SNPs and ITP, we performed a TaqMan PCR genotyping assay. Genotypic frequencies in patients and controls were in the Hardy-Weinberg equilibrium (*P* > 0.05, Supplementary [Supplementary-material supplementary-material-1]). The top 4 SNPs except for rs4483616 due to failure of probe synthesis were genotyped. Strong association of these SNPs with ITP were observed (*P* < 0.05, Table 2). The minor allelic frequency (MAF) of rs5998634 C/T genotype in ITP cases was significantly a higher risk than in controls (14.4% versus 4.5%, *P* = 0.0019, OR = 3.19, 95%CI = 1.98–5.16). Both the distributions of rs117503120 G/A and rs16866133 T/G in ITP patients were significantly lower than in controls (6% versus 14.0%, *P* = 0.026, OR = 0.43, 95%CI = 0.27–0.67 and 0.5% versus 6.0%, *P* = 0.03, OR = 0.20, 95%CI = 0.08–0.49, respectively).

### 3.4. SNPs Associated with ITP Patient Response to Glucocorticoid Therapy

**Figure 4:**
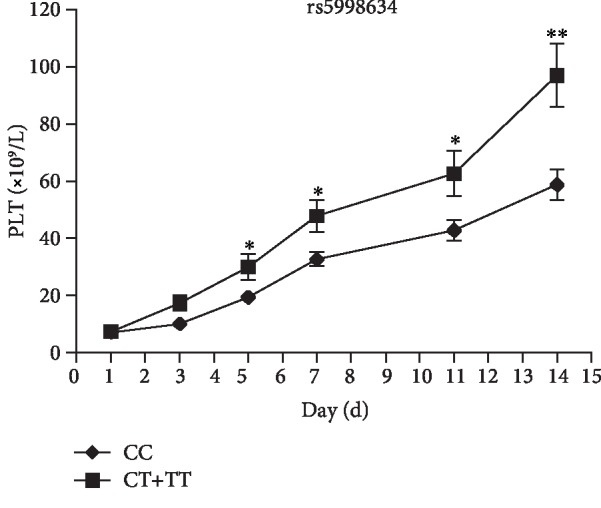
Association of the allele T of rs5998634 with the change of platelet count focus on the ITP patients treated with glucocorticoid. The mean platelet count of 148 ITP patients with CC genotype (●) and 35 patients with CT or TT genotype (■) was calculated after treated with glucocorticoid at different time points: 1, 3, 5, 7, 11, and 14 days. ^∗∗^*P* < 0.01, ^∗^*P* < 0.05. Data represent mean ± SD. (*n* = 148 in CC genotype group, *n* = 35 in CT or TT genotype group).

We compared the genotype and allele frequencies of rs117503120, rs5998634, and rs16866133 between the response and nonresponse group for glucocorticoid therapy. The results showed that only the rs5998634 minor allele T was significantly associated with a favorable response to glucocorticoid treatment among ITP patients (*χ*^2^ = 7.30, *P* = 0.03) (Table 3). In term of rs5998634, there were 148 ITP patients with CC genotype and 35 patients with CT or TT genotype. We further evaluated the change of platelet count focused on the different genotypes of rs5998634 for ITP patients after the treatment of glucocorticoid within two weeks. As shown in Figure 4, the mean platelet count of ITP patients with the rs5998634 CT or TT genotype was significantly higher than in patients with the CC genotype after the fifth day of glucocorticoid treatment (*P* < 0.05).

### 3.5. Pathways Involved in the Etiology of ITP

We identified three potentially candidate pathways from the KEGG database: JAK-STAT signaling pathway (Adj − *P* = 0.0054), neuroactive ligand-receptor interaction (Adj − *P* = 0.0054), and pathways in cancer (Adj − *P* = 0.0099) (Table 4, Supplementary Figures [Supplementary-material supplementary-material-1]).

## 4. Discussion

The genetic factor plays a nonnegligible role in modulating the course of ITP. Due to the low incidence of ITP, genetic analysis with large sample sizes can be challenging. Traditional candidate gene approaches have identified the relationship of genes such as IL-10, IL-3, and IFN-*λ* with ITP; but these findings have not elucidated the genetic etiology of ITP at a whole-genome scale [15, 16]. To extend the present genetic evidence and to identify novel candidates of ITP, we performed a GWAS combining with pathway analysis for ITP from CHP. Our results revealed that 4 novel loci of *GBE1*/*LINC02027* (rs117503120), *TIMP3*/*SYN3* (rs5998634), *TENM4* (rs4483616), and *RBM45*/*OSBPL6* (rs16866133) were strongly associated with ITP from CHP.

Rs117503120 is located on the *GBE1*/*LINC02027* gene at chromosome 3q12.3. The glucan (1,4-alpha-) branching enzyme 1 (*GBE1*) was reported strongly associated with glycogen storage disease in previous research [17]. Our GWAS results showed that *GBE1* was significantly associated with ITP (*P* = 6.45 × 10^−9^), and the TaqMan probe genotyping results also showed that the MAF of rs117503120 in ITP cases was significantly lower than controls, suggesting that the rs117503120 minor allele may be protective for ITP. Thus, *GBE1* may have a potential vital association with ITP from CHP, although the mechanism is yet to be investigated. In addition, the *LINC02027* (long intergenic nonprotein coding RNA 2027) has been reported to be highly expressed in liver and kidney tissues from the 95 human individuals [18], but the detailed relationship with ITP remains unclear.

Rs5998634 is located on *TIMP3*/*SYN3* at chromosome 22q12.3, which was strongly associated with ITP in our study (*P* = 8.06 × 10^−8^). The tissue inhibitor of metalloproteinase-3 (TIMP3) is an inhibiting matrix metalloproteinase protein [19]. Researches have shown that the TIMP-3 protein has a statistically positive correlation with IL-4 and platelet count, but a negative correlation with IFN-*γ* in ITP patients, suggesting that this protein may lead to Th1/Th2 polarization via affected antigen-presenting cells and contribute to the occurrence and development of autoimmune disease [20]. Importantly, the TaqMan probe genotyping results confirmed such a strong association of rs5998634 with ITP (*χ*^2^ = 12.5, *P* = 0.0019). The MAF of rs5998634 CT genotype in ITP patients (0.144) was significantly higher than in controls (0.045), suggesting that the T allele is a major genetic risk factor to ITP from CHP. Furthermore, we also evaluated the change difference of platelet count from the 183 ITP patients treated with glucocorticoid based on the different genotypes of rs5998634. Interestingly, the mean platelet count of 35 ITP patients with the rs5998634 CT or TT genotype was significantly higher than in 148 patients with the CC genotype after the fifth day of glucocorticoid treatment (*P* < 0.05). These results shown that the patients with ITP who carry the rs5998634 T allele may be more sensitive to glucocorticoids than patients with the C allele. The limited sample size and lack of replication resulted in low statistical efficiency, and follow-up studies with large sample sizes need to be carried out. Synapsin III (*SYN3*) mainly involved in the development of brain or neurons disease, such as Parkinson disease [21], but the detailed relationship with ITP remains to be confirmed.

Rs16866133 is located on *RBM45*/*OSBPL6* at chromosome 2q31.2 and also had a strong association with ITP in our study (*P* = 9.39 × 10^−8^). The association of RNA-binding motif protein 45 (*RBM45*) with ITP or other autoimmune diseases is unknown. The oxysterol-binding protein-like 6 (*OSBPL6*) gene encodes the oxysterol-binding protein-like 6 receptor, which associated with multiple sclerosis (*P* = 4.64 × 10^−4^) in the United Kingdom (UK) population [22]. It is not a surprise that ITP shares some immune mechanisms with multiple sclerosis; thus, *OSBPL6* may participate in the pathogenesis of ITP in terms of immune regulation. Also, the TaqMan probe genotyping results showed a significantly lower MAF of rs16866133 TG genotype in ITP patients compared with the controls (0.005 vs 0.060), suggesting that rs16866133 G was a protective allele for ITP.

To provide further insight into the molecular function of identified associated variants, the pathway analysis converged GWAS datasets was conducted. In this study, the JAK-STAT signaling pathway, neuroactive ligand-receptor interaction, and pathways in cancer were proposed to be the most potentially associated with ITP from CHP. Among them, the JAK-STAT signaling pathway has been previously reported to participate in the etiological mechanism of pediatric ITP using the gene expression profile analysis methods [23]. It also plays a major role in the pharmacological mechanisms of eltrombopag, which is a thrombopoietin (TPO) receptor agonist approved by the FDA for the treatment of chronic ITP patients [24]. Thus, our results further provide evidence that the JAK-STAT signaling pathway involved in the pathogenesis of ITP, but its detail mechanism still needed to be explored. However, neuroactive ligand-receptor interaction and pathways in cancer have not been extensively studied in ITP to date. These findings motivate an in-depth evaluation of the contribution of these loci and pathways in the etiology of ITP.

## Figures and Tables

**Table 1:** Clinical characteristic of ITP patients and controls.

Population	Age (year)	Gender, female (%)	WBC (×10^9^/L)	RBC (×10^12^/L)	HB (g/L)	PLT (×10^9^/L)
Stage 1						
Cases (*n* = 200)	40.36 ± 16.39	135 (67.5)	8.72 ± 4.53	3.95 ± 0.79	114.33 ± 25.31	16.10 ± 27.22
Controls (*n* = 200)	40.78 ± 16.03	135 (67.5)	6.57 ± 1.60	4.39 ± 0.36	131.18 ± 11.41	207.36 ± 50.34
*P*			<0.001	<0.001	<0.001	<0.001
Stage 2						
Cases (*n* = 250)	40.85 ± 12.21	168 (67.2)	8.46 ± 4.72	3.68 ± 0.90	105.96 ± 26.46	24.57 ± 35.04
Controls (*n* = 250)	41.63 ± 12.99	168 (67.2)	5.86 ± 1.68	4.27 ± 0.37	134.00 ± 11.31	201.96 ± 53.35
*P*			<0.001	<0.001	<0.001	<0.001

WBC, white blood cell; RBC, red blood cell; HB, hemoglobin; PLT, platelet count. The significance threshold is *P* value <0.05.

**Table 2:** The genotype frequency of four significant loci between ITP patients and controls.

SNP	Closest genes	Chr	Position	*P* value in pooling GWAS	Risk allele	Genotypes	Genotype frequency		*χ* ^2^	OR (95% CI)	*P*
							Cases *n* (%)	Controls *n* (%)			
rs117503120	GBE1/LINC02027	3	81352557	6.45 × 10^−9^	G	GG	235 (94.0)	215 (86.0)	7.28	0.43 (0.27–0.67)	0.026
						GA	15 (6.0)	35 (14.0)			
						AA	0 (0.0)	0 (0.0)			

rs4483616	TENM4	11	78824323	8.84 × 10^−9^	/	/	/	/	/	/	/
					/	/	/	/	/	/	/
					/	/	/	/	/	/	/

rs5998634	TIMP3/SYN3	22	33169115	8.06 × 10^−8^	T	CC	212 (84.8)	238 (95.2)	12.5	3.19 (1.98–5.16)	0.0019
						CT	36 (14.4)	12 (4.8)			
						TT	2 (0.8)	0 (0.0)			

rs16866133	RBM45/OSBPL6	2	179022610	9.39 × 10^−8^	T	TT	247 (99.5)	235 (94.0)	7.73	0.20 (0.08–0.49)	0.03
						TG	3 (0.5)	15 (6.0)			
						GG	0 (0.0)	0 (0.0)			

SNP, single nucleotide polymorphism; Chr, chromosome; ITP, primary immune thrombocytopenic; CI, confidence interval; the positions are based on the GRCh37 assembly. The significance threshold is *P* value <1.0 × 10^−7^.

**Table 3:** The genotype distribution of rs117503120, rs5998634, and rs16866133 between the glucocorticoid response group and nonresponse group.

SNP	Closest genes	Risk allele	Genotypes	Genotype frequency		*χ* ^2^	*P*
				Response *n* = 120	Nonresponse *n* = 63		
rs117503120	GBE1/LINC02027	G	GG	114	62	0.49	0.78
			GA	6	1		
			AA	0	0		

rs5998634	TIMP3/SYN3	T	CC	89	59	7.30	0.03
			CT	30	4		
			TT	1	0		

rs16866133	RBM45/OSBPL6	T	TT	119	63	0.04	0.98
			TG	4	0		
			GG	0	0		

The significance threshold is *P* value <0.05.

**Table 4:** The overrepresentation pathways identified by the top 287 genes with a *P* value <1.0 × 10^−5^ in this pooling GWAS.

Pathway name	Total	Observed	*E*-ratio	*P*	Adj-*P*
JAK-STAT signaling pathway	155	6	6.27	0.0004	0.0054
Neuroactive ligand-receptor interaction	256	8	5.06	0.0002	0.0054
Pathways in cancer	330	8	3.93	0.0011	0.0099

The number of reference genes in the category (total); ratio of enrichment (*E*-ratio); *P* value from hypergeometric test; *P* value adjusted by the multiple test adjustment (Adj-*P*), the significant threshold of Adj-*P* is *P* < 1.0 × 10^−5^.

## Data Availability

The data used to support the findings of this study are available from the Gene Expression Omnibus as data set GSE76744 (https://www.ncbi.nlm.nih.gov/geo/query/acc.cgi?acc=GSE76744).

## Supplementary Materials

Supplementary Materials Supplementary Table 1: The FPRP of the top 20 loci in the pooling GWAS of ITP. Supplementary Table 2: Other SNPs in linkage disequilibrium to the rs117503120, rs4483616, rs5998634, and rs16866133. Supplementary Table 3: Hardy-Weinberg equilibrium analysis of the genotype frequencies of rs117503120, rs5998634, and rs16866133. Supplementary Figures. Supplementary Figure 1: The flowchart of two stage sample collection. Supplementary Figure 2: Scatter plot of pooling genotypic data. Predicted allele frequencies of 862,620 randomly selected SNPs in ITP case and control DNA pools, *r*^2^ = 0.9947. Supplementary Figure 3: KEGG pathway for the JAK-STAT signaling pathway. The significant genes (JAK2, IL12A, TPO, SPRY2, CBLB, and CSF3R) in our GWAS are shown in red. Supplementary Figure 4: KEGG pathway for the neuroactive ligand-receptor interaction signaling pathway. The significant genes (PTGER3, NPY2R, MCHR2, GRIK3, GABRG2, OPRM1, CCKAR, GRM8, and CSF3R) in our GWAS are shown in red. Supplementary Figure 5: KEGG pathway for the pathway in cancer pathway. The significant genes (FGF9, COL4A2, CBLB, PTCH1, ETS1, TCF7L2, CSF3R, and FAS) in our GWAS are shown in red.
