# Supplementary material for: A Pooling Genome-Wide Association Study Identifies Susceptibility Loci and Signaling Pathways of Immune Thrombocytopenia in Chinese Han Population
Source: Int J Genomics. 2020 Mar 11;2020:7531876. doi: 10.1155/2020/7531876 (PMC7086454; doi:10.1155/2020/7531876)
Supplement: Supplementary Materials — Supplementary Table 1: The FPRP of the top 20 loci in the pooling GWAS of ITP. Supplementary Table 2: Other SNPs in linkage disequilibrium to the rs117503120, rs4483616, rs5998634, and rs16866133. Supplementary Table 3: Hardy-Weinberg equilibrium analysis of the genotype frequencies of rs117503120, rs5998634, and rs16866133. Supplementary Figures. Supplementary Figure 1: The flowchart of two stage sample collection. Supplementary Figure 2: Scatter plot of pooling genotypic data. Predicted allele frequencies of 862,620 randomly selected SNPs in ITP case and control DNA pools, r2 = 0.9947. Supplementary Figure 3: KEGG pathway for the JAK-STAT signaling pathway. The significant genes (JAK2, IL12A, TPO, SPRY2, CBLB, and CSF3R) in our GWAS are shown in red. Supplementary Figure 4: KEGG pathway for the neuroactive ligand-receptor interaction signaling pathway. The significant genes (PTGER3, NPY2R, MCHR2, GRIK3, GABRG2, OPRM1, CCKAR, GRM8, and CSF3R) in our GWAS are shown in red. Supplementary Figure 5: KEGG pathway for the pathway in cancer pathway. The significant genes (FGF9, COL4A2, CBLB, PTCH1, ETS1, TCF7L2, CSF3R, and FAS) in our GWAS are shown in red. [file 7531876.f1.pdf]

Supplementary Table 1. The FPRP of the top 20 loci in the pooling GWAS of ITP.

Nearest Gene	SNP	Chr	Position	FPRP	*P*-value	OR	95% CI
GBE1/ LINC02027	rs117503120	3	813,52,557	0.012435	6.45×10^{-9}	7.24	3.53-14.8
TENM4	rs4483616	11	78,824,323	0.012025	8.84×10^{-9}	5.82	3.08-11.01
SYN3/TIMP3	rs5998634	22	33,169,115	< 0.00001	8.06×10^{-9}	0.35	0.25-0.48
RBM45/OSBPL6	rs16866133	2	179,022,610	0.026704	9.39×10^{-8}	0.2	0.11-0.36
RGS1/RGS13	kgp4981410	1	192,572,631	0.00605	1.44×10^{-7}	2.27	1.7-3.04
DNAH17	rs8069561	17	76,451,921	0.058062	3.07×10^{-7}	0.27	0.17-0.45
LOC100289378/HS1BP3	rs921598	2	20,712,253	0.032657	4.38×10^{-7}	2.51	1.78-3.55
SPATA8/LOC91948	rs4523904	15	98,047,823	0.008292	4.98×10^{-7}	0.38	0.27-0.54
REEP3	rs16918582	10	65,304,328	0.007242	6.69×10^{-7}	2.85	1.96-4.14
LRP1B	kgp7502028	2	142,417,595	0.149265	7.39×10^{-7}	2.85	1.87-4.31
LOC643496/INTRON	kgp8967884	2	168,209,246	0.002475	8.65×10^{-7}	0.35	0.24-0.50
MVP	kgp16485512	16	29,851,738	0.169019	1.11×10^{-6}	0.43	0.31-0.61
CST4/CST1	rs13044018	20	23,692,121	0.082799	1.21×10^{-6}	0.45	0.61-0.59
NGR3/LOC728027	rs17101655	10	85,050,535	0.011547	1.22×10^{-6}	2.46	1.77-3.41
CRYBB3/CRYBB2	rs4820621	22	25,608,548	0.158445	1.26×10^{-6}	2.38	1.68-3.81
LRP1B/INTRON	rs11898305	2	142,423,793	0.073087	1.26×10^{-6}	4.21	2.41-7.34
MDC1/TUBB	rs9295910	6	30,687,714	0.000114	1.30×10^{-6}	0.37	0.27-0.50
SNTG2/TPO	rs17091595	2	1,405,849	0.078586	1.63×10^{-6}	2.87	1.90-4.33
MPHOSPH6/CDH13	rs4408537	16	82,408,872	0.107083	1.88×10^{-6}	0.32	0.21-0.50
PTPRD	rs10977936	9	9,734,267	0.036741	1.98×10^{-6}	4.17	2.43-7.15

FPRP: false-positive report probabilities; OR: Odds ratio.

Supplementary Table 2. Other SNPs in linkage disequilibrium to the rs117503120, rs4483616, rs5998634, and rs16866133.

uniqID	rsID	Chr	Pos	non_effect_allele	effect_allele	MAF	gwasP	r^2	IndSigSNP
3:81352557:A:G	rs117503120	3	81352557	A	G	0.05357	6.45E-09	1	rs117503120
11:78824323:A:G	rs4483616	11	78824323	A	G	0.04663	8.84E-09	1	rs4483616
11:78823774:C:T	rs12292238	11	78823774	T	C	0.04663	NA	1	rs4483616
11:78827407:A:G	rs61882093	11	78827407	G	A	0.04663	NA	1	rs4483616
11:78828322:C:T	rs7930419	11	78828322	C	T	0.04663	NA	1	rs4483616
11:78834003:C:T	rs948762	11	78834003	C	T	0.04563	NA	0.978595	rs4483616
11:78870881:C:T	rs503601	11	78870881	C	T	0.04563	NA	0.604768	rs4483616
22:33169115:C:T	rs5998634	22	33169115	T	C	0.07143	8.06E-08	1	rs5998634
22:33168562:A:G	rs5994625	22	33168562	G	A	0.07143	NA	1	rs5998634
22:33170101:C:T	rs135150	22	33170101	T	C	0.07143	NA	1	rs5998634
22:33170683:C:T	rs130531	22	33170683	C	T	0.07143	NA	1	rs5998634
22:33175471:C:G	rs130535	22	33175471	G	C	0.07143	NA	1	rs5998634
22:33148102:C:T	rs73156461	22	33148102	C	T	0.04663	NA	0.631038	rs5998634
22:33148391:A:G	rs78194905	22	33148391	G	A	0.04663	NA	0.631038	rs5998634
22:33155017:A:G	rs1018791	22	33155017	G	A	0.04365	NA	0.620738	rs5998634
22:33158361:C:T	rs129303	22	33158361	C	T	0.04464	NA	0.633507	rs5998634
22:33162023:A:G	rs129305	22	33162023	G	A	0.04464	NA	0.633507	rs5998634
22:33162672:C:T	rs130526	22	33162672	C	T	0.04464	NA	0.633507	rs5998634
22:33163549:A:G	rs130527	22	33163549	A	G	0.04464	NA	0.633507	rs5998634
22:33165252:C:T	rs130528	22	33165252	T	C	0.04464	NA	0.633507	rs5998634
22:33165464:A:C	rs130529	22	33165464	C	A	0.04464	NA	0.633507	rs5998634
22:33173055:C:G	rs135151	22	33173055	C	G	0.04464	NA	0.633507	rs5998634

2:179022610:G:T	rs16866133	2	179022610	G	T	0.02083	9.39E-08	1	rs16866133
2:179021928:A:G	rs7569572	2	179021928	A	G	0.02083	NA	1	rs16866133
2:179022730:G:T	rs62176040	2	179022730	T	G	0.01984	NA	0.950413	rs16866133
2:179031809:A:C	rs16866146	2	179031809	C	A	0.01885	NA	0.901031	rs16866133
2:179047132:A:G	rs62177185	2	179047132	A	G	0.01885	NA	0.901031	rs16866133
2:179051111:G:T	rs192290900	2	179051111	G	T	0.01389	NA	0.657143	rs16866133
2:179054976:C:T	rs62177188	2	179054976	T	C	0.01687	NA	0.707337	rs16866133
2:179061637:A:G	rs60829693	2	179061637	A	G	0.01687	NA	0.707337	rs16866133
2:179063979:C:T	rs62177210	2	179063979	C	T	0.01687	NA	0.707337	rs16866133

uniqID: Unique ID of SNPs consisting of chr:position:allele1:allele2 where alleles are alphabetically ordered.
rsID: rsID of the lead SNPs;
Chr: chromosome;
Pos: genomic position (hg19);
MAF: Micro Allele Frequency
gwasP: the P-value of GWAS
IndSigSNP: rsID of independent significant SNPs which are in LD with the lead SNP at r^2 0.1.

Supplementary Table 3. Hardy-Weinberg equilibrium analysis of the genotype frequencies of rs117503120, rs5998634, and rs16866133.

SNP	Genotypes	Cases		χ^2	*P*	Controls		χ^2	*P*
		Observed	Expected			Observed	Expected		
rs117503120	GG	235	235.2			215	216.2		
	AG	15	14.6	0.24	0.62	35	32.6	1.42	0.23
	AA	0	0.2			0	1.2		
rs5998634	CC	212	211.6			238	238.1		
	CT	36	36.8	0.12	0.73	12	11.7	0.15	0.70
	TT	2	1.6			0	0.1		
rs16866133	TT	247	247.0			235	235.2		
	GT	3	3.0	0.009	0.92	15	14.6	0.24	0.62
	GG	0	0.0			0	0.2		

The $P > 0.05$ was considered to be consistent with the Hardy-Weinberg equilibrium.

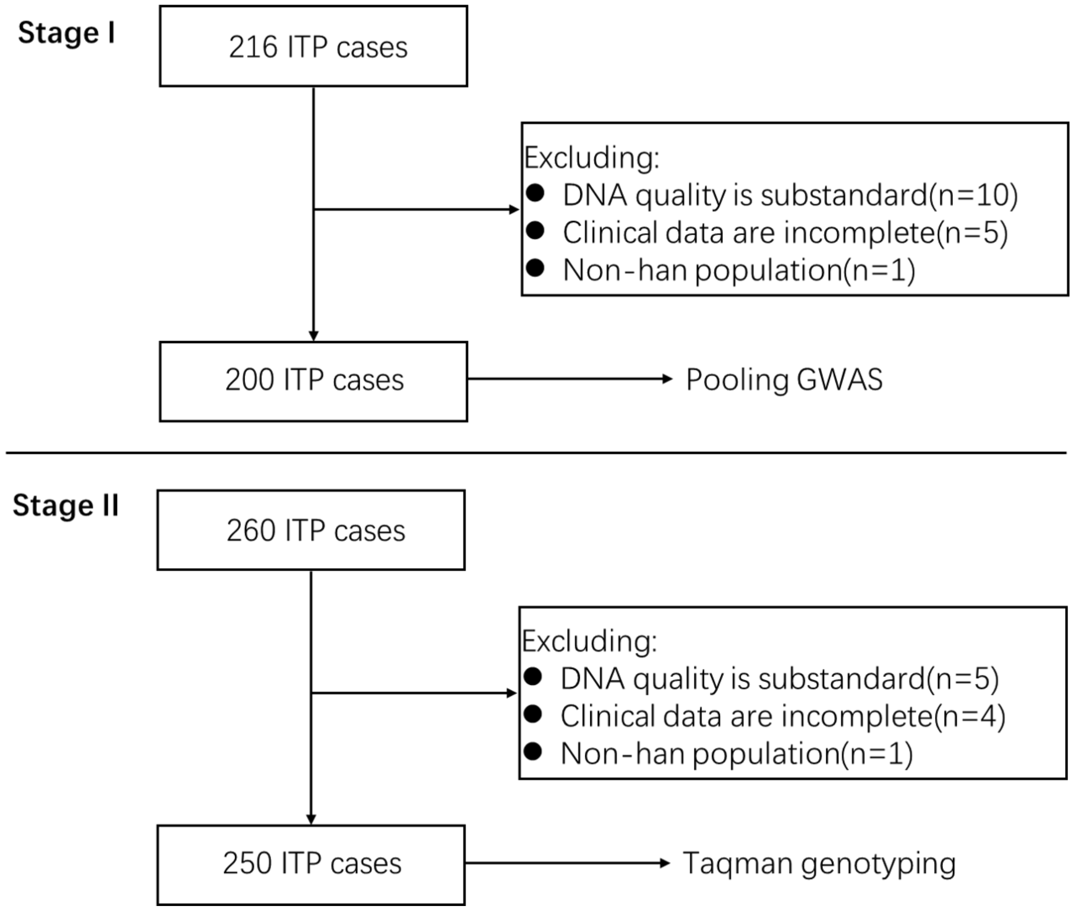

Supplementary Figure 1: The flowchart of two stage sample collection.

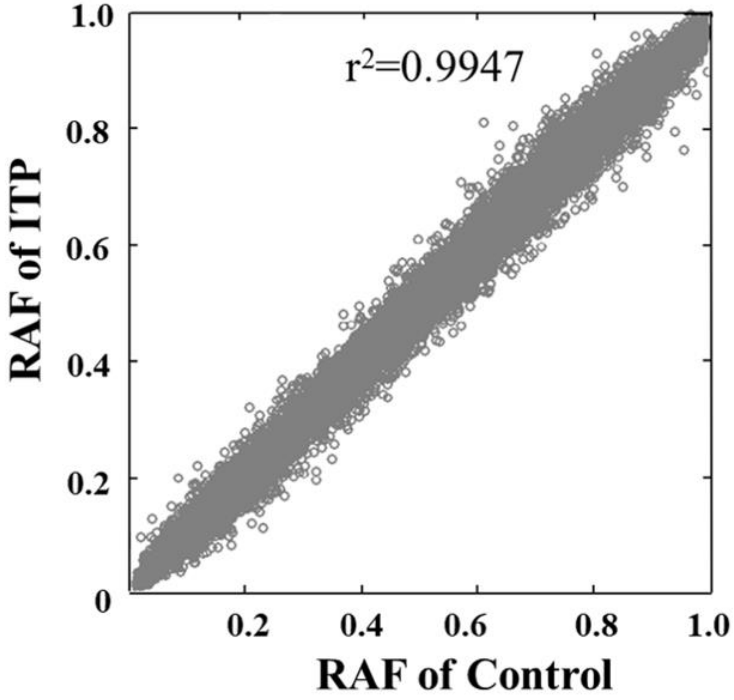

Supplementary Figure 2: Scatter plot of pooling genotypic data. Predicted allele frequencies of 862,620 randomly selected SNPs in ITP case and control DNA pools, $r^2 = 0.9947$. Abbreviations: ITP: Immune thrombocytopenia; SNPs, single-nucleotide polymorphisms.

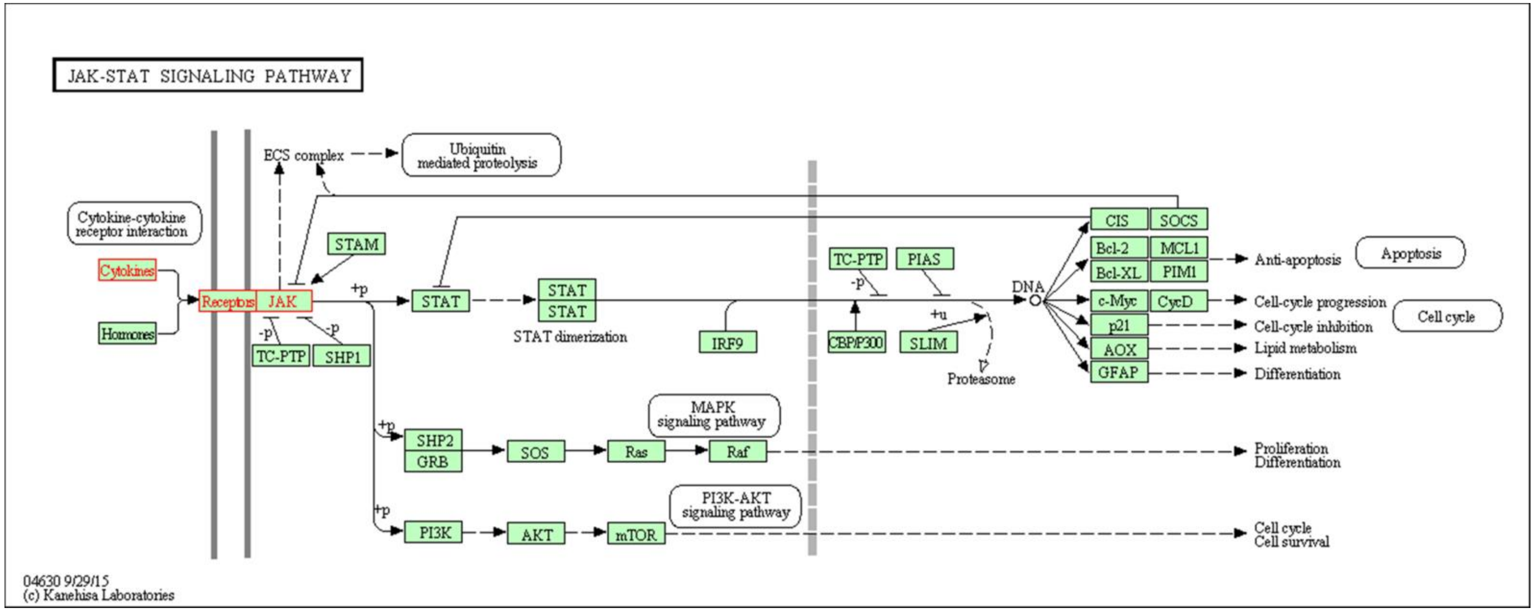

Supplementary Figure 3.

KEGG pathway for the JAK-STAT signaling pathway. The significant genes (*JAK2*, *IL12A*, *TPO*, *SPRY2*, *CBLB*, and *CSF3R*) in our GWAS are shown in red.

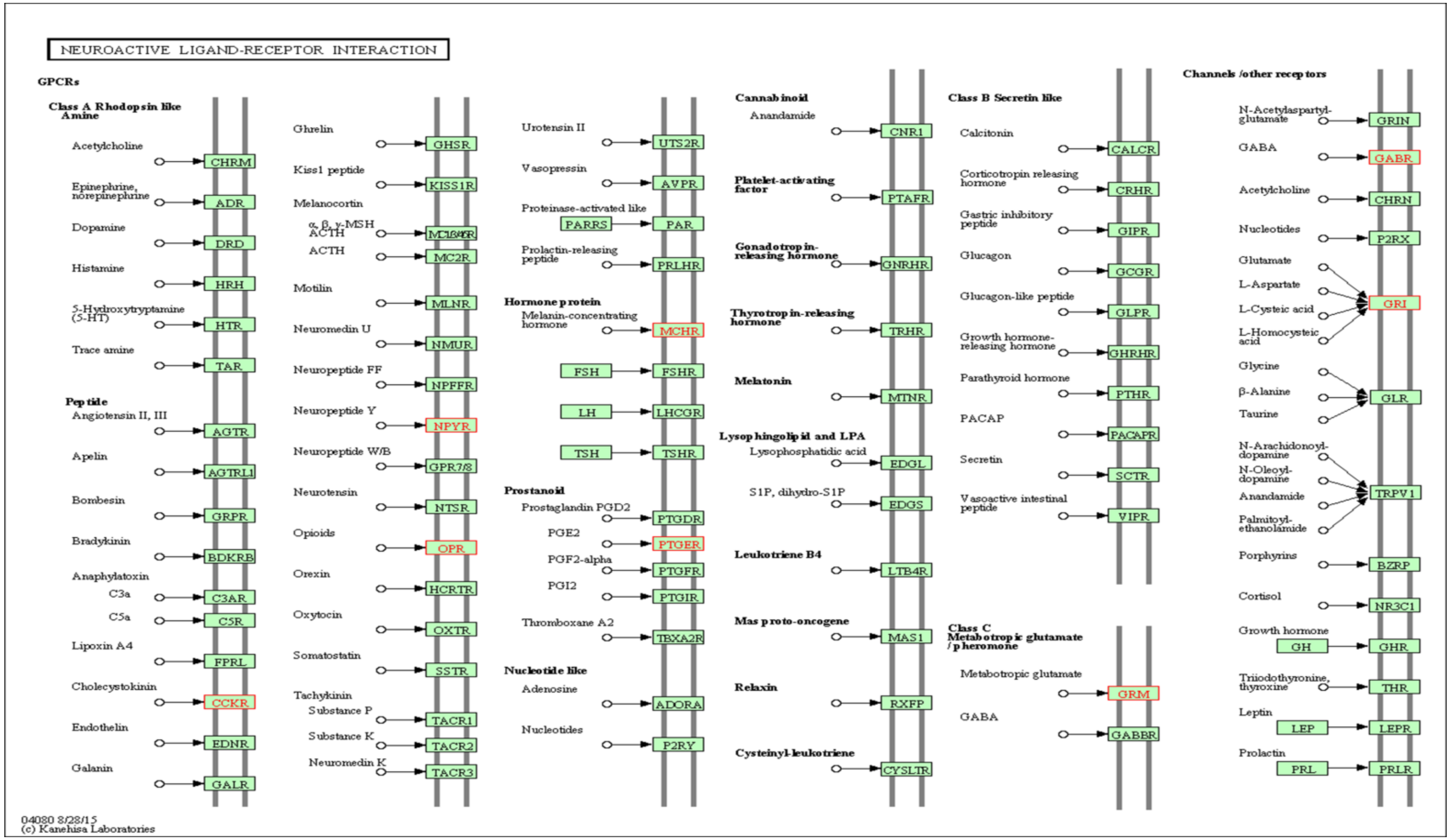

Supplementary Figure 4.

KEGG pathway for the Neuroactive ligand-receptor interaction signaling pathway. The significant genes (PTGER3, NPY2R, MCHR2, GRIK3, GABRG2, OPRM1, CCKAR, GRM8, and CSF3R) in our GWAS are shown in red.

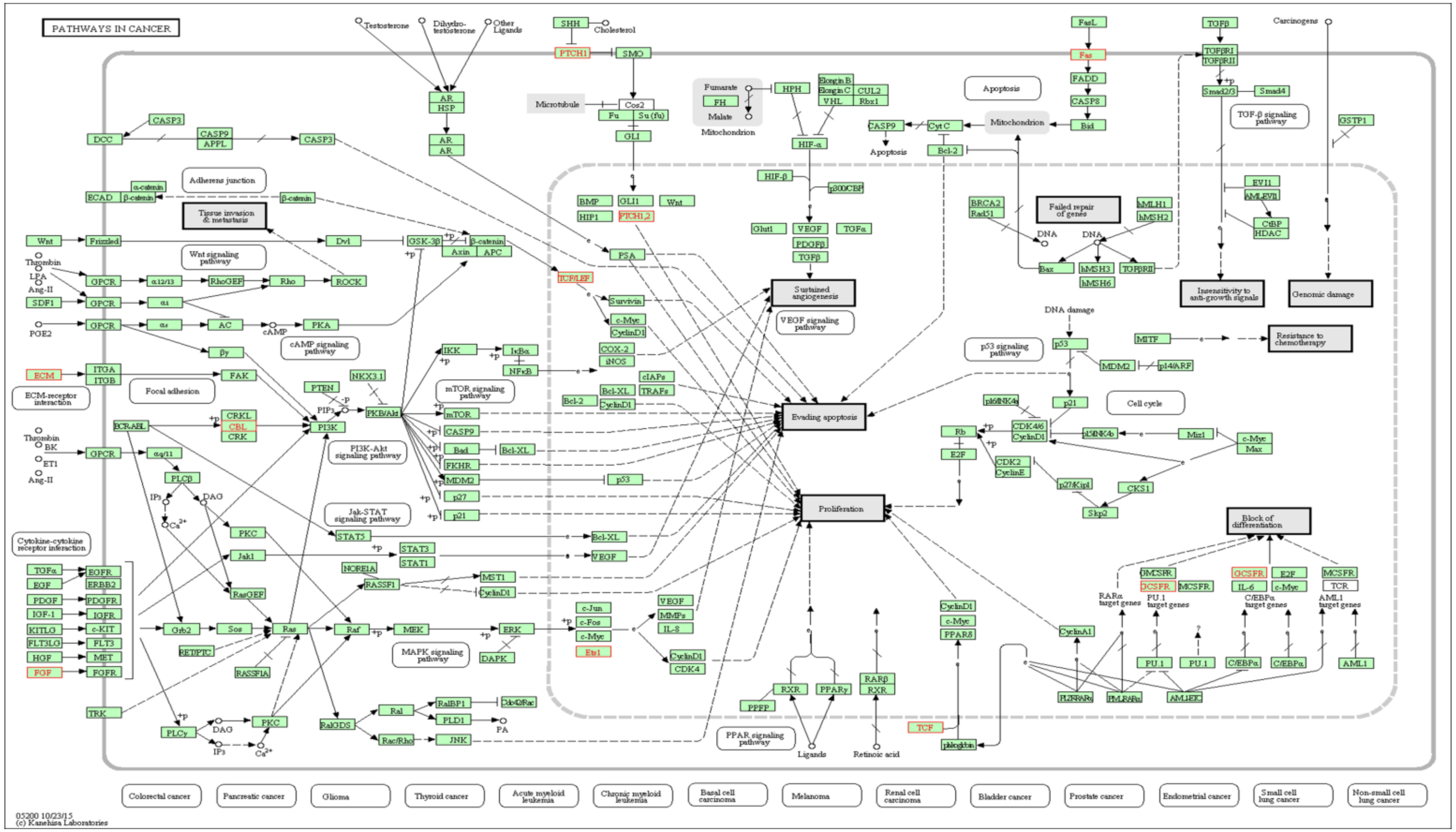

Supplementary Figure 5.

KEGG pathway for the Pathway in cancer pathway. The significant genes (*FGF9*, *COL4A2*, *CBLB*, *PTCH1*, *ETS1*, *TCF7L2*, *CSF3R*, and *FAS*) in our GWAS are shown in red.
